# A Hypoglycemic Peptide from *Pinus pumila* Nut Oil Meal Improves Glycolipid Metabolism via Multi-Dimensional Regulation in Type 2 Diabetic Mice

**DOI:** 10.3390/nu17172903

**Published:** 2025-09-08

**Authors:** Zhe-Xuan Mu, Zhen-Zhou Li, Bing-Xiao Liu, Zhen-Yu Wang, Xiao-Hong Lv, Lin Yang, Hua Zhang

**Affiliations:** 1 Department of Special Food and Drug and Biochemical Innovation Research Center, School of Chemistry and Chemical Engineering, Harbin Institute of Technology, Harbin 150001, China; 23s025127@stu.hit.edu.cn (Z.-X.M.); 23s025129@stu.hit.edu.cn (Z.-Z.L.); m18845618140@163.com (B.-X.L.); wzy219001@163.com (Z.-Y.W.); 2 Forestry and Grassland Germplasm Resources Research Center, Heilongjiang Academy of Forestry, Harbin 150081, China; 18645105533@163.com

**Keywords:** *Pinus pumila* bioactive peptides, agricultural by-product utilization, glycolipid metabolism regulation, gut microbiota modulation, KEGG pathway analysis

## Abstract

Background and Methods: To address the need for dietary interventions in sub-healthy populations and promote sustainable utilization of agricultural by-products, we isolated *Pinus pumila* hypoglycemic peptide (PHP) from nut oil meal through enzymatic extraction, ion exchange and gel chromatography purification, and simulated gastric digestion. Results: PHP exhibited significant inhibitory activity against α-amylase and α-glucosidase. In type 2 diabetic mice, PHP significantly ameliorated the “three-more-one-less” syndrome, reduced glycosylated hemoglobin and insulin levels, mitigated liver and kidney tissue lesions, and improved glucose and lipid metabolic disorders—effects partly supported by its enhancement of intestinal barrier function via restoring gut microbiota diversity. Gut microbiota analysis revealed that PHP exerts hypoglycemic effects by regulating gut microbial composition: increasing SCFA-producing taxa, reducing pro-inflammatory/metabolic disorder-associated taxa, and normalizing the Firmicutes/Bacteroidetes ratio. KEGG pathway analysis demonstrated that PHP mediates synergistic hypoglycemic effects by regulating carbohydrate metabolism, amino acid metabolism, and cofactor/vitamin metabolism. Conclusions: This work provides a theoretical foundation for developing natural functional foods from agricultural by-products, supporting PHP’s potential as a dietary supplement for metabolic regulation.

## 1. Introduction

In its November 2024 report, the World Health Organization (WHO) disclosed that the global adult diabetic population has surpassed 800 million, highlighting the imperative for coordinated global interventions (https://www.who.int/). In the United States, 90–95% of diabetes cases are obesity-associated type 2 diabetes mellitus (T2DM), compounded by a worrying rise in adolescent overweight/obesity rates—one-third of American youth now fall into the overweight category [1]. Despite the extensive clinical use of synthetic agents such as metformin, pioglitazone, and teneligliptin, their therapeutic application is frequently hindered by side effects including abdominal pain, headache, upper respiratory tract infections, and heart failure, coupled with efficacy limitations stemming from their single-target mechanisms of action [2]. Acarbose, a clinically employed semisynthetic α-glucosidase inhibitor, effectively controls postprandial glycemia with a better safety profile than synthetic drugs but may cause mild intestinal adverse effects [3]. Natural plant extracts have emerged as promising alternatives for diabetes management, distinguished by their low/non-toxicity, high biocompatibility, and multi-target synergistic effects compared to conventional small-molecule drugs [4]. Hypoglycemic peptides have been isolated from various crops, such as beans, rice [5,6,7,8,9]. However, the resource development of tree nuts (e.g., pine nuts) remains underexplored. Notably, secondary metabolites of Pinaceae species are typically exhibit anti-inflammatory and antioxidant activities, suggesting their extracts may possess potential for modulating glucose metabolism.

*Pinus pumila* (Pall.) Regel, native to cold-zone and subalpine regions spanning northeastern China, the Russian Far East, and the Korean Peninsula, produces seeds rich in highly unsaturated fatty acids and high-quality proteins [10]. Its nut oil meal, a by-product with >30% protein content, is currently predominantly used as animal feed, with limited systematic exploration of its bioactive components. Plant by-products are known sources of diverse bioactive peptides (e.g., antioxidant, hypolipidemic, and hypotensive). Enzymatically extracted peptides exhibit advantages including low molecular weights, enhanced bioavailability, and superior stability across pH and temperature gradients [11]. In vitro screening of hypoglycemic peptides typically evaluates their inhibitory effects on α-glucosidase and α-amylase [12]. Studies have shown that the inhibitory activity of plant-derived peptides against these two enzymes increases significantly following gastrointestinal digestion [13]. This is likely due to pepsin-mediated selective hydrolysis, which releases low-molecular-weight active fragments and enhances peptide affinity for enzyme active sites by exposing hydrophobic residues [14]. In vivo, researchers widely use type 2 diabetic mouse models to verify the peptide-mediated regulatory mechanisms of glucose and lipid metabolism, where lipid metabolism status is assessed using lipid metabolic factors such as cholesterol levels, and glucose metabolic function via key rate-limiting enzymes like hexokinase [15]. Intestinal microbiota research represents a frontier in elucidating the mechanisms of metabolic diseases [16], with ginseng peptides, for example, enhancing glucose metabolism by regulating intestinal flora dysbiosis and short-chain fatty acid (SCFA) metabolism [17].

In this study, novel peptides were extracted, purified, and characterized from *Pinus pumila* nut oil meal, aiming to address its underutilization. We base this study on the scientific community’s long-standing exploration of extracting bioactive substances from agricultural by-products, thereby advancing the deep-processing technology for pine-derived by-products; concurrently, this approach retains practical significance for supporting the development of the circular economy.

## 2. Materials and Methods

### 2.1. Materials

*Pinus pumila* nuts (Ning’an Beiyu Zhenqi Shanlin Food Co., Ltd., Ningan, China); Papain (Meryer (Shanghai) Chemical Technology Co., Ltd., Shanghai, China); Alkaline protease, streptozotocin (STZ), α-glucosidase, p-nitrophenyl β-D-galactopyranoside (PNPG) (Macklin Biochemical Technology Co., Ltd., Shanghai, China); Diethylaminoethyl-cellulose anion exchange column (DEAE-Cellulose 52), Sephadex™ G50 medium, phosphate buffered saline (PBS), dimethylguanidine hydrochloride (Solarbio Science & Technology Co., Ltd., Beijing, China); Simulated gastric fluid (SGF) (Phygene Biotechnology Co., Ltd., Fuzhou, China); α-Amylase (Shanghai Yuanye Bio-Technology Co., Ltd., Shanghai, China); Mouse feed (Jiangsu Synergy Biotechnology Co., Ltd., Nanjing, China); Petroleum ether, NaCl, Na_2_CO_3_, soluble amylose, 3,5-dinitrosalicylic acid, glucose, polyformaldehyde (Sinopharm Group Co., Ltd., Beijing, China); Glycated haemoglobin kit, pyruvate kinase kit, hexokinase kit, phosphofructokinase kit, triglyceride kit, total cholesterol kit, HDL cholesterol kit, LDL cholesterol kit, hepatoglycogen/myoglycogen test kit, glucose-6-phosphate dehydrogenase kit (Nanjing Jiancheng Bioengineering Institute, Nanjing, China); Mouse ADP (adiponectin) ELISA kit, mouse INS (insulin) ELISA kit, mouse LEP (leptin) ELISA kit (KELU (Wuhan) Biotechnology Co., Ltd., Wuhan, China); Quant-iT™ PicoGreen^®^ dsDNA assay kit (Thermo Fisher Scientific Inc., Waltham, MA, USA); TruSeq™ Nano DNA LT Library Prep Kit (Illumina, Inc., San Diego, CA, USA).

### 2.2. Preparation of Crude Extracts of Peptides from Pinus pumila Nut

The protein extraction from *Pinus pumila* nuts was executed using an experimental protocol optimized in our prior study [18]. Distilled water (1:50 *w*/*v*) was added to the extracted proteins, and the mixture was extracted for 4 h at pH 9.0 (measured with a PHS-3E pH meter, Shanghai Yidian Scientific Instrument Co., Shanghai, China) and 50 °C (using a DF-101 magnetic stirrer, Gongyi Huayi Machinery Factory, Gongyi, China), with the addition of 6000 U g^−1^ alkaline protease, where U g^−1^ here denotes the enzyme activity added per gram of substrate. After a 15 min boiling water bath for enzyme inactivation, the mixture was centrifuged at 6790× *g* for 20 min (H2050R benchtop high-speed freezing centrifuge, Hunan Xiangyi Experimental Instrument Development Co., Ltd., centrifuge, Hunan Xiangyi Experimental Instrument Development Co., Ltd., Changsha, China). The supernatant was adjusted to neutral pH, dialyzed for 48 h (1000 Da cutoff), and freeze-dried (LGJ-50F freeze dryer, Beijing Songyuan Huaxing Technology Development Co., Ltd., Beijing, China) to yield the crude *P. pumila* nut peptide extract.

### 2.3. Determination of Peptide Crude Extract Hydrolysis

The hydrolysis degree of peptide crude extracts was calculated using Formulas (1) and (2) [19]:

(1) $$H=B\times N_{b}\times 1/\alpha \times 1/M_{a\;}$$

where B—volume of NaOH solution consumed during hydrolysis (mL); N_b_—concentration of NaOH solution (mol L^−1^); Ma—mass of protein in hydrolysate (g); and α—calibration coefficients (varying with temperature and pH).

(2) $$DH=H/H_{hot}\times 100\%$$

where H—amount of hydrolyzed peptide bonds per unit protein mass (mmol g^−1^); H_hot_—total peptide bonds per unit protein mass (mmol g^−1^), a fixed value for specific proteins (taken as 7.335).

### 2.4. Purification of Pinus pumila Peptide Crude Extracts

Primary purification was performed using DEAE-Cellulose 52 under the following separation conditions: a 1.0 cm diameter column with 30 cm height and eluent flow rate of 2.0 mL min^−1^ (collected by an SBS-100 automatic partial collector, Shanghai Biochemical Instrument Co., Ltd., Shanghai, China) at 3 min per tube. A 2.0 mL sample (20 mg mL^−1^) was loaded, and elution was carried out with distilled water, 0.1 M NaCl, 0.3 M NaCl, and 0.5 M NaCl—each eluent volume was four times the column volume. Secondary purification was performed using Sephadex™ G-50 Medium under the following conditions: a 3 cm diameter column (40 cm height) was loaded with 5 mL of sample (20 mg mL^−1^), eluted at 1.25 mL/min (4 mL per tube) with distilled water. The elution volume was four times the column volume, and Fraction 2 containing low-molecular-weight (high-absorption) peptides were collected [20].

### 2.5. Simulated Gastric Digestion of Fraction 2

Fraction 2 was digested with simulated gastric fluid (SGF, 1:100 *w*/*v*) for 80 min, inactivated by boiling water bath for 15 min, and centrifuged at 3820× *g* for 15 min, and the supernatant was dialyzed (200 Da) for 48 h followed by freeze-drying to obtain *Pinus pumila* hypoglycemic peptide (PHP). Peptide content in PHP was determined using the Fonkwe and Singh method [21].

### 2.6. α-Glucosidase Inhibition by PHP

#### 2.6.1. Inhibition Rate Determination

Experimental conditions were modified slightly based on preliminary results [22]: 100 μL PHP solutions (0.01–100.00 mg mL^−1^) were mixed with 50 μL α-glucosidase solution (1.25 U mL^−1^) and 50 μL p-nitrophenyl β-D-glucopyranoside (PNPG, 8.0 mmol L^−1^), incubated for 20 min, and terminated with 100 μL Na_2_CO_3_ (1.0 mol L^−1^). The α-glucosidase inhibition rates of PHP at different concentrations were calculated based on the absorbance measured at 405 nm using an Infinite M200 pro microplate reader (Tecan Group Ltd., Zurich, Canton of Zurich, Switzerland), and the *IC_50_* value was fitted.

#### 2.6.2. Reversibility Analysis of PHP’s Inhibitory Effect on α-Glucosidase

With fixed substrate concentration (8.0 mmol L^−1^ PNPG), α-glucosidase (0.00–1.75 U/mL) and PHP (0.0–7.5 mg/mL) concentrations were varied. The remaining procedures were performed as described for the inhibition rate determination to assess the reversibility of PHP-mediated α-glucosidase inhibition [23].

#### 2.6.3. Inhibition Type Analysis

With fixed α-glucosidase (1.25 U mL^−1^), PNPG (0–8 mmo L^−1^) and PHP (0.0–7.5 mg/mL) concentrations were varied. Kinetic parameters (Km and Vmax) were determined to characterize inhibition type, following the same protocols as inhibition rate assays [24].

### 2.7. α-Amylase Inhibition by PHP

#### 2.7.1. Inhibition Rate Determination

Experimental conditions were modified slightly based on preliminary results [25]: reactions contained 30 μL α-amylase (2 U mL^−1^), 40 μL PHP (0.01–100.00 mg mL^−1^), and 30 μL 2% (*w*/*v*) soluble starch. Following 10 min incubation at 37 °C, 100 μL of the DNS reagent was added to terminate the reaction. The α-amylase inhibition rates of PHP at different concentrations were calculated based on the absorbance measured at 540 nm using an Infinite M200 pro microplate reade, and the *IC_50_* value was fitted.

#### 2.7.2. Reversibility Analysis of PHP’s Inhibitory Effect on α-Amylase

The substrate concentration (2% soluble starch) was fixed, the concentration of α-amylase solution (0.0–5.0 mg mL^−1^) and the concentration of PHP solution (0.0–7.5 mg mL^−1^) were varied, and the remaining steps were carried out in the same way as for the determination of the inhibition rate, and it was assessed whether the PHP inhibition of α-amylase was reversible or not [26].

#### 2.7.3. Inhibition Type Analysis

At a fixed α-amylase concentration (2.0 U mL^−1^), reactions were performed with varying soluble starch (0.1–0.5% *w*/*v*) and PHP (0.0–7.5 mg mL^−1^) concentrations. Kinetic parameters (Km and Vmax) were determined to characterize inhibition type, following the same protocols as inhibition rate assays [27].

### 2.8. PHP Sequence Identification

The primary amino acid sequence of PHP was determined by liquid chromatography–tandem mass spectrometry (LC-MS/MS) [28]. Isolated fractions were analyzed by mass spectrometry in positive-ion mode using a Q Exactive HF-X Mass Spectrometer (Thermo Fisher Scientific, Waltham, MA, USA).

### 2.9. T2DM Mouse Modeling and Grouping

SPF-grade male KM mice (25 ± 2 g, Liaoning Changsheng Biotechnology, Benxi, China; SCXK(Liao)2020-0001) were adaptively fed for 1 week. Ten mice were randomly selected as the normal control (NC) group, fed a standard diet (60% carbohydrate, 20% protein, and 20% fat) with free access to food and water. The remaining 90 mice underwent T2DM modeling: after 30 days on a high-fat diet (HFD; 20% carbohydrate, 60% fat, and 20% protein), they were fasted for 12 h and intraperitoneally injected with 1% STZ (60 mg kg^−1^ body weight) on three consecutive days [29]. Mice with fasting blood glucose (FBG) of 11.1–25.0 mmol L^−1^ (measured by Roche Glucose Meter, Roche Diagnostics GmbH, Mannheim, Germany) 1 week later were considered successfully modeled [30]. T2DM mice were randomized into five groups (10 mice/group) for 4-week gavage treatment: T2DM control: 0.3 mL 0.9% saline; MET: 75 mg kg^−1^ metformin hydrochloride; PHPL: 75 mg kg^−1^ PHP; PHPM: 125 mg kg^−1^ PHP; and PHPH: 250 mg kg^−1^ PHP [17,31,32,33]. T2DM mice were allocated to groups using a fully randomized number table. Cages were randomly positioned in the housing facility. Data collection and gavage were performed in a blinded sequence, with only the statistician overseeing randomization aware of group assignments.

### 2.10. Effects of PHP on Basal Physiological Indices in T2DM Mice

During the gavage treatment, food intake was monitored every 3 days, water intake every 2 days, and fasting body weight with FBG every 5 days (12-h fast, *n* = 10 per group). On day 29 of treatment, fasted mice underwent oral glucose tolerance testing (OGTT): blood glucose was measured at 0, 30, 60, and 90 min post-glucose gavage, with area under the curve (AUC) calculated from tolerance curves [34].

### 2.11. Effect of PHP on Biochemical Indices in T2DM Mice

Blood samples were collected via retro-orbital puncture from 10 mice per experimental group and centrifuged at 9700× *g* for 15 min (DragonLab D2012 Plus benchtop centrifuge, Beijing DragonLab Scientific Instruments Co., Ltd., Beijing, China), and supernatants (serum) were stored at −80 °C. Lipid metabolism markers [triglycerides (TG), total cholesterol (TC), high-density lipoprotein (HDL), low-density lipoprotein (LDL), adiponectin (ADPN), and leptin (LEP)], glucose metabolic enzymes [hexokinase (HK), phosphofructokinase (PFK), pyruvate kinase (PK), and glucose-6-phosphate dehydrogenase (G6PD)], and glucose homeostasis indices [glycosylated hemoglobin and insulin (INS)] were assayed. Insulin resistance was evaluated by HOMA-IR, calculated from fasting blood glucose (FBG) and INS levels [35].

### 2.12. Effect of PHP on Organ Tissue Morphology in T2DM Mice

Liver and kidney tissues were collected from 3 mice per experimental group, rinsed with 0.9% saline, fixed in 4% paraformaldehyde, dehydrated through gradient ethanol (50–100%), paraffin-embedded, and sectioned into 6 μm thick cross-sections. These sections were stained with hematoxylin–eosin (HE) and analyzed by white-light panoramic scanning using a digital pathology scanner (KF-PRX-040, Zhejiang Jiangfeng Bio Information Technology, Shanghai, China) under optical microscopy.

### 2.13. Effect of PHP on Intestinal Flora of T2DM Mice

Fresh fecal samples were subjected to DNA extraction, quantified by spectrophotometry, and diluted to 1 ng μL^−1^. The V3-V4 hypervariable region of the 16S rRNA gene was amplified using specific primers, with products verified by 1.2% agarose gel electrophoresis. Amplicons were purified via magnetic bead-based recovery, quantified by fluorescence (FLx800 Microplate Reader, BioTek, Agilent Technologies, Inc., Santa Clara, CA, USA), and used to construct Illumina sequencing libraries. Paired-end sequencing (2 × 250 bp) was performed on a NovaSeq 6000 platform (Illumina, Inc., San Diego, CA, USA), followed by KEGG pathway analysis of 16S rRNA sequencing data.

### 2.14. Statistical Analysis

All experimental data were derived from three independent replicates and presented as mean ± standard deviation. GraphPad Prism 9 (GraphPad Software, Boston, MA, USA) and Origin 2024 (OriginLab, Northampton, MA, USA) were used for data visualization: bar graphs for conventional kit assays and box plots for ELISA results. Differences between groups were analyzed using one-way analysis of variance (one-way ANOVA). For post hoc multiple comparisons, the least significant difference (LSD) test was applied to data with homogeneous variances. Meanwhile, with the model group as the reference, Dunnett’s test (two-tailed) was used to compare experimental groups with the reference group. Statistical description and homogeneity of variance test were performed prior to statistical analysis. A *p*-value < 0.05 was considered statistically significant (IBM SPSS Statistics 27, IBM, Armonk, NY, USA).

## 3. Results and Discussion

### 3.1. Graded Purification of Crude Extracts of Pinus pumila Peptides

**Figure 1:**
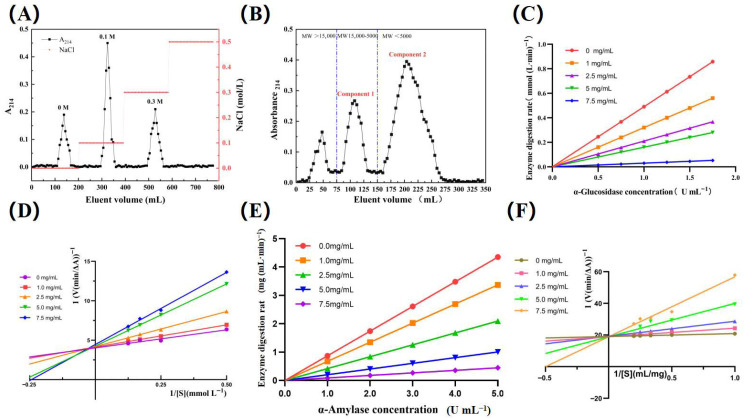
(**A**) Ion exchange chromatography purification elution curve; (**B**) gel chromatography purification elution curve; (**C**) inhibition type of PHP on α-glucosidase; (**D**) inhibition kinetics of PHp on a α-glucosidase; (**E**) inhibition type of PHP on α-amylase; (**F**) inhibition kinetics of PHP on α-amylase.

During hydrolysis, 1.415 mL of 2 mol L^−1^ NaOH was consumed (correction factor, α = 1.01; pH = 9.0, 50 °C), resulting in a 25.10% degree of hydrolysis for the crude peptide extract. This value is substantially higher than that reported for *Tenebrio molitor* peptides [36], suggesting potential superior biological activity and enhanced absorbability. The crude *Pinus pumila* peptide extract bound reversibly to DEAE-Cellulose 52 via electrostatic interactions. Gradient elution with NaCl yielded three purified fractions, each displaying excellent symmetry and purity (no tailing) as shown in Figure 1A [34]. Notably, the 0.3 M NaCl-eluted fraction—with a yield of approximately 15% (relative to the protein)—exhibited significantly stronger inhibitory activity against α-amylase and α-glucosidase than other fractions. This phenomenon arises from two effects: low salt concentrations disrupt peptide–enzyme electrostatic interactions, while high concentrations induce peptide aggregation, both reducing enzyme inhibitory capacity. The 0.3 M NaCl fraction was therefore selected for subsequent gel column purification.

As shown in Figure 1B, Sephadex™ G-50 gel column chromatography fractionated the 0.3 M NaCl eluate by molecular weight [37]. Fraction 2—eluted with distilled water—was selected for enrichment based on its high peptide content, low molecular weight, and freedom from small-molecule impurities or macromolecular aggregates, and a yield of 8–10% (relative to the protein). Its markedly stronger enzyme inhibitory activity stems from three key features: the short amino acid chains of low-molecular-weight peptides, their flexible spatial conformations, and the ready exposure of hydrophobic residues. These properties enable Fraction 2 to form stronger affinity with the enzyme active site via hydrogen bonding and hydrophobic interactions. Additionally, low-molecular-weight fragments have a larger specific surface area, allowing binding to more enzyme sites per unit mass and thereby enhancing inhibition efficiency [38]. Fraction 2 was further digested with simulated gastric fluid (SGF) to potentiate its hypoglycemic activity, yielding a purified product termed PHP with a peptide content of 82.75% ± 0.86% and a yield of approximately 6%. Impurities in PHP stem from three sources: enzyme-released phenolic-peptide complexes [39]; raw material-derived residual oils and fat-soluble pigments, unremovable during purification due to poor water solubility; and unseparated oligosaccharide fragments with peptide-like molecular weights. In vitro inhibitory activity data from the purification process and gastric digestion are summarized in Table 1.

### 3.2. Sequence Identification of PHP

To ensure rigorous structural identification, LC-MS/MS analysis was performed on both the Fraction 2 and PHP. The top 10 high-confidence sequences from each sample were selected and cross-validated via sequence, leading razor protein, and protein name analyses. As shown in Table 2, we identified Fraction 2 as the peptide sequence SDDVLEAAFNTDVQKLEHIFGAH and PHP as NTDVQKLEHIFGAH by classifying leading razor proteins and screening for shared peptides (Table S1). This confirms specific cleavage at the Phe9-Asn10 (F9-N10) site by simulated gastric fluid (SGF) digestion, consistent with pepsin’s preference for cleaving aromatic amino acids at the P1 position [40]. PHP purity, determined via peak intensity analysis of high-confidence sequences (score > 200), was 73.92% [41]. PHP’s acidic residues (Asp3 (D) and Glu8 (E)) bind to positively charged residues (Arg/Lys/His) in the α-amylase/α-glucosidase active centers via hydrogen bonds and salt bridges [42]. Conversely, its basic residues (Lys6 (K) and His13 (H)) competitively block enzyme catalytic interfaces (e.g., the α-amylase catalytic triad) and inhibit substrate hydrolysis through charge shielding, yielding a synergistic hypoglycemic effect [43].

### 3.3. Mixed Inhibition of α-Glucosidase by PHP

As a key enzyme in terminal glucose hydrolysis, α-glucosidase regulates glucose absorption in the intestinal tract by modulating its own activity to control postprandial blood glucose levels. The half-maximal inhibitory concentration (*IC_50_*) of PHP for α-glucosidase was 5.21 ± 0.14 mg mL^−1^, significantly lower than that of ginkgo biloba seed protein (*IC_50_* = 12.94 mg mL^−1^) [44] and comparable to hot-pressed peanut meal protein (*IC_50_* = 5.63 mg mL^−1^) [45]. As shown in Figure 1C, all kinetic curves were linear through the origin, with slopes decreasing progressively as PHP concentration increased, indicating that PHP inhibits α-glucosidase activity without altering enzyme concentration (slope: 0.49, 0.32, 0.21, 0.16, 0.03) [46]. This reversible inhibition suggests non-covalent binding of PHP to the enzyme. Figure 1D shows that increasing PHP concentration decreases Vmax and increases Km, confirming mixed inhibition [47]. This suggests that PHP is capable of binding to both the free enzyme and enzyme–substrate complex—analogous to non-competitive inhibition—yet exhibits a greater affinity for the free enzyme, akin to the competitive inhibition.

### 3.4. Competitive Inhibition of α-Amylase by PHP

A-Amylase functions as an endoglycosidase, hydrolyzing starch α-1,4-glycosidic bonds to yield oligosaccharides. Inhibition of its activity delays proximal intestinal glucose release, while modulating insulin sensitivity and glucagon secretion to regulate postprandial glucose levels. The IC_50_ of PHP for α-amylase was 1.71 ± 0.08 mg mL^−1^, comparable to germinated soybean peptides (*IC_50_* = 1.70 mg mL^−1^) [48], slightly lower than *Pisum sativum* L. protein hydrolysate (*IC_50_* > 4.2 mg mL^−1^) [49], and significantly superior to pea protein peptides (*IC_50_* = 6.75 mg mL^−1^) [50]. As shown in Figure 1E, PHP exhibited a dose-dependent linear inhibition of α-amylase, mirroring the inhibition pattern of α-glucosidase with reversible characteristics. Figure 1F shows that increasing PHP concentration significantly elevates Km while Vmax remains unchanged, with reaction system lines intersecting on the vertical axis—kinetic hallmarks of competitive inhibition. Consistent with competitive inhibitor traits, PHP likely shares structural similarity with the substrate, specifically recognizing and competing for the enzyme’s active site; binding forms an enzyme–inhibitor complex, precluding substrate association [51].

### 3.5. Effects of PHP on Basal Physiological Indices in T2DM Mice

**Figure 2:**
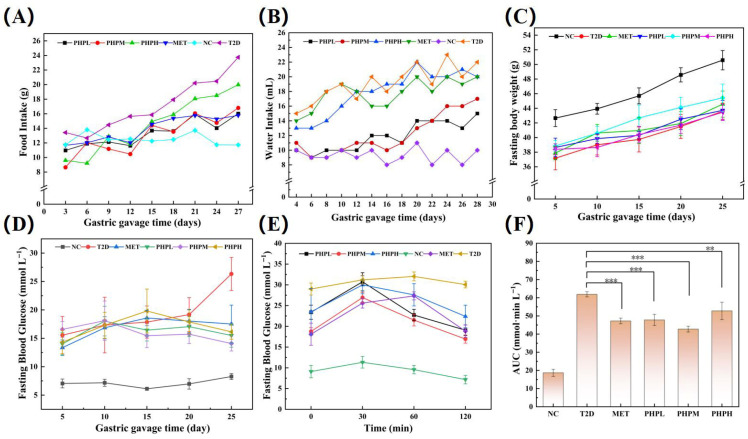
Effect of PHP on basal physiological indexes in T2DM mice: (**A**) food intake; (**B**) water intake; (**C**) fasting body weight; (**D**) fasting blood glucose; (**E**) oral glucose tolerance curve; (**F**) area under the oral glucose tolerance curve (AUC). Difference was significant at ** *p* < 0.01 and *** *p* < 0.001.

As shown in Figure 2A and Figure 2B, the NC group maintained stable daily food (12 g) and water (9 mL) intake, while the T2DM group displayed marked hypermetabolism: food (24 g) and water (20 mL) intake were 2.0- and 2.2-fold higher than the NC group, respectively. PHP treatment mitigated polydipsia and polyphagia in T2DM mice, though intake remained elevated relative to the NC group.

As shown in Figure 2C, fasting body weights increased synchronously across all groups (non-significant difference). Despite this parallel growth, both the T2DM and PHP intervention groups had significantly lower final body weights than the NC group, attributable to their lower initial masses. As shown in Figure 2D, the NC group maintained stable FBG at 8.0 mmol L^−1^, significantly lower than other groups (>13.0 mmol L^−1^). During gavage, the T2DM group showed persistent FBG elevation to 27.0 mmol L^−1^, whereas the PHP group showed significantly reduced FBG after 15 days of intervention. As shown in Figure 2E,F, PHP’s ameliorative effect on glucose tolerance mirrored the activity of selenium polysaccharides reported previously [52]. In the PHP-treated group, blood glucose peaked at 30 min and then declined continuously to baseline by 120 min, with a significant reduction in OGT area. These findings suggest that PHP may exert hypoglycemic effects by enhancing glucose metabolism or insulin sensitivity.

### 3.6. Effect of PHP on Lipid Metabolism in T2DM Mice

#### 3.6.1. Effect of PHP on Blood Lipid Profiles in T2DM Mice

**Figure 3:**
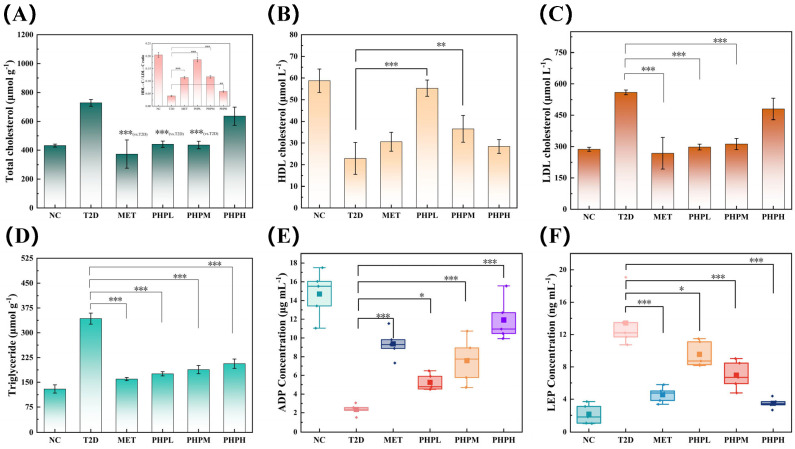
Effect of PHP on lipid metabolism in T2DM mice: (**A**) total cholesterol level; (**B**) high-density cholesterol level; (**C**) low-density cholesterol level; (**D**) triglyceride level; (**E**) adiponectin level; (**F**) leptin level. Difference was significant at * *p* < 0.05, ** *p* < 0.01, and *** *p* < 0.001.

T2DM mice induced by high-fat diet (HFD) combined with streptozotocin (STZ) consistently exhibit pathological features of glycolipid metabolic disturbance [53]. As shown in Figure 3A–D, the T2DM group showed significant elevations in total cholesterol (TC, +68.91%), triglycerides (TG, +143.97%), and low-density lipoprotein cholesterol (LDL-C, +95.13%), alongside a 61.03% reduction in high-density lipoprotein cholesterol (HDL-C) compared to the NC group.

All PHP-treated groups demonstrated restorative effects on dysregulated lipid parameters in T2DM mice. The TC levels in the PHPL group did not differ significantly from those in the NC and MET groups. This finding is consistent with existing research conclusions demonstrating that MET ameliorates hypercholesterolemia [54]. Among PHP dose groups, PHPM exhibited the most balanced lipid-lowering efficacy. Similar to the effects of peanut flour peptides on lipid profiles, PHP displayed dose-dependent improvements at lower concentrations; like pea oligopeptides, higher PHP doses may induce antagonistic effects impeding recovery [55,56]. The PHPL group exhibited a 48.22% lower TG content than the T2DM group, though levels remained significantly distinct from the MET group [57]. The HDL content in the PHP group increased to the level of the NC group and was superior to that of the MET group [58]. LDL levels in PHPL were significantly lower than in T2DM (46.83% reduction) and comparable to MET, consistent with the lipid—regulating properties of most plant-derived hypoglycemic peptides [59,60]. PHP matched MET in reducing total cholesterol and LDL, but outperformed it in restoring HDL. This indicates that PHP has a unique ability to ameliorate lipid metabolism disorders.

#### 3.6.2. Effect of PHP on Lipid Metabolism Regulators in T2DM Mice

T2DM mouse models induced by HFD combined with STZ exhibit key biological features: reduced levels of adiponectin (ADPN, a marker of hepatic gluconeogenesis) and elevated levels of leptin (LEP, a marker of insulin resistance) [61]. As shown in Figure 3E,F, compared to the NC group, the T2DM group exhibited an 83.23% decrease in ADPN and a 264.06% increase in LEP. PHP restored levels of adipose metabolic factors in a dose-dependent manner: in the PHPH group, it restored ADPN to 78.10% of NC levels (with no significant difference compared to MET), while decreasing LEP by 72.63% relative to the T2DM group, slightly outperforming MET. These findings indicate that PHP contributes to alleviating glucose metabolic disorders.

### 3.7. Effect of PHP on Glucose Metabolism in T2DM Mice

#### 3.7.1. Effect of PHP on the Glycolytic Pathway in T2DM Mice

**Figure 4:**
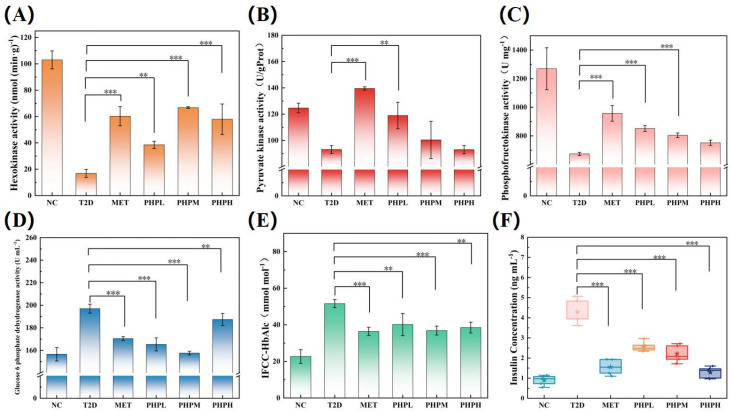
Effect of PHP on glucose metabolism in T2DM mice: (**A**) hexokinase activity; (**B**) pyruvate kinase activity; (**C**) phosphofructokinase activity; (**D**) glucose 6 phosphate dehydrogenase activity; (**E**) glycated hemoglobin level; (**F**) insulin level. Difference was significant at ** *p* < 0.01 and *** *p* < 0.001.

In the glycolytic pathway, hexokinase (HK), phosphofructokinase (PFK), and pyruvate kinase (PK) catalyze three irreversible reactions to regulate energy production: HK initiates the phosphorylation of glucose to glucose-6-phosphate (G6P); PFK, a rate-limiting enzyme, converts fructose-6-phosphate to fructose-1,6-bisphosphate; and PK converts phosphoenolpyruvate to pyruvate, generating ATP to complete the cycle. Collectively, these enzymes determine glycolytic flux, and their dysfunction correlates with impaired glucose metabolism in T2DM. As shown in Figure 4A,B,C, hepatic HK, PFK, and PK activities in T2DM mice were significantly reduced by 83.64%, 46.89%, and 25.00%, respectively, indicating overall inhibition of the pathway. PHP intervention significantly upregulated all three enzymes (HK: +312.50%, PFK: +26.26%, PK: +27.76%), with HK showing the most pronounced activation—likely by relieving G6P-mediated feedback inhibition to prevent excessive glucose consumption [62]. The significant regulation of PFK suggests involvement of the AMPK pathway or insulin signaling [63]. PHP’s ability to co-upregulate all rate-limiting enzymes may contribute to maintaining glucose homeostasis, potentially through enhanced islet sensitivity and AMPK pathway activation [64].

#### 3.7.2. Effect of PHP on the Pentose Phosphate Pathway (PPP) in T2DM Mice

Glucose-6-phosphate dehydrogenase (G6PD), the rate-limiting enzyme of the PPP, catalyzes G6P oxidation to ribulose-5-phosphate and NADPH, the cell’s primary reducing agent [65]. Unlike type 1 diabetes, HFD-STZ-induced T2DM mice exhibit significantly elevated G6PD activity (25.82% increase here) versus NC mice [66], a compensatory response to oxidative stress. Sustained hyperglycemia overloads the mitochondrial electron transport chain, elevates reactive oxygen species (ROS), and upregulates G6PD to produce NADPH for redox homeostasis. As shown in Figure 4D, PHP normalized G6PD activity in the PHPM group to the level observed in the NC group (with no significant difference) and outperformed metformin (MET). These results indicate that PHP, like MET, contributes to maintaining glycemic stability.

#### 3.7.3. Effect of PHP on Glycemic Homeostasis in T2DM Mice

Glycated hemoglobin (HbA1c), formed via non-enzymatic glycation of hemoglobin, reflects mean glycemia over 30 days. As shown in Figure 4E, the T2DM group had a 127.43% higher HbA1c level (48.0–53.0 mmol mol^−1^) than the NC group, indicating the mice were in the early stages of diabetes. At this stage, compensatory glucose elevation is maintained through increased insulin secretion or activation of the PPP (e.g., upregulated G6PD activity) [67]. This aligns with our experimental findings: insulin levels were significantly elevated (3.8-fold higher than in the NC group) alongside insulin resistance, as depicted in Figure 4F. All PHP doses significantly reduced HbA1c in T2DM mice, with efficacy comparable to that of MET. Insulin secretion in the PHPH group was markedly decreased, reaching levels similar to MET and approaching those of the NC group. These findings indicate that PHP may reverse the compensatory hyperinsulinemic phase of early diabetes by enhancing peripheral insulin sensitivity (e.g., in muscle) and mitigating oxidative stress-mediated β-cell damage [68].

### 3.8. Effect of PHP on Hepatic and Renal Morphology in T2DM Mice

**Figure 5:**
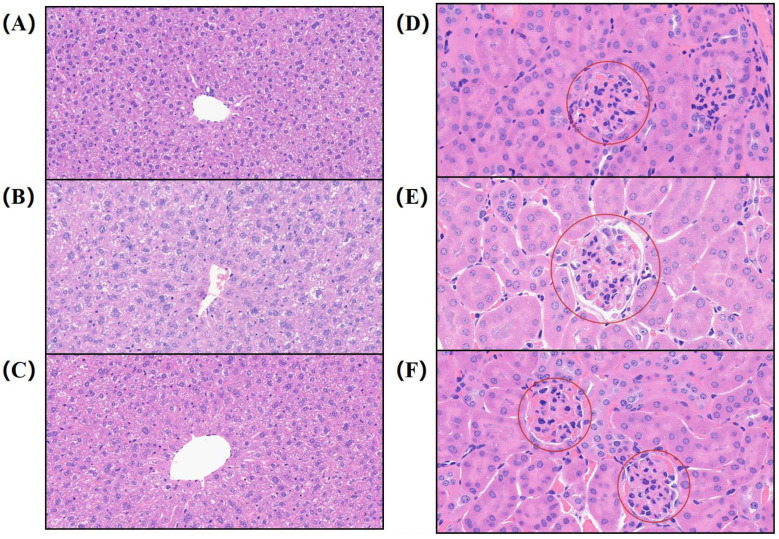
Morphologic analysis of liver: (**A**) NC group; (**B**) T2DM group; (**C**) PHPM group. Morphologic analysis of kidney: (**D**) NC group; (**E**) T2DM group; (**F**) PHPM group. The red circle represents a glomerulus.

A high-fat diet (HFD) combined with STZ injection typically induces severe organ damage in mice. As shown in Figure 5A–C, livers from T2DM mice appeared pale pink compared to those from NC controls, indicating reduced hepatic glycogen deposition. Histological analysis revealed hepatocyte hyalinization, hemocyte aggregation in the central vein, marked dilatation of hepatic sinusoids, blurred boundaries of liver plates, hepatocyte cytoplasmic vacuolization, and partial hepatocyte steatosis. Additionally, T2DM mice exhibited a significant reduction in the number of hepatic Kupffer cells, suggesting impaired liver function. PHP effectively reduced central vein inflammatory cell infiltration and improved hepatic sinusoid dilatation, similar to the effect of spexin [69]. As shown in Figure 5D–F, compared to NC controls, T2DM mice exhibited renal pathological changes: glomerular hypertrophy, dilated cystic lumens, thickened glomerular capillary basement membrane (GBM), expanded mesangial matrix, narrowed vascular lumens, detachment and edema of tubular epithelial cells, interstitial fibrosis, and inflammatory cell infiltration. These changes are morphologically consistent with those in other diabetic models. PHP alleviated cystic lumen enlargement and significantly improved cellular edema and glomerular hypertrophy.

### 3.9. Effect of PHP on Intestinal Flora in T2DM Mice

#### 3.9.1. Species Composition Analysis

**Figure 6:**
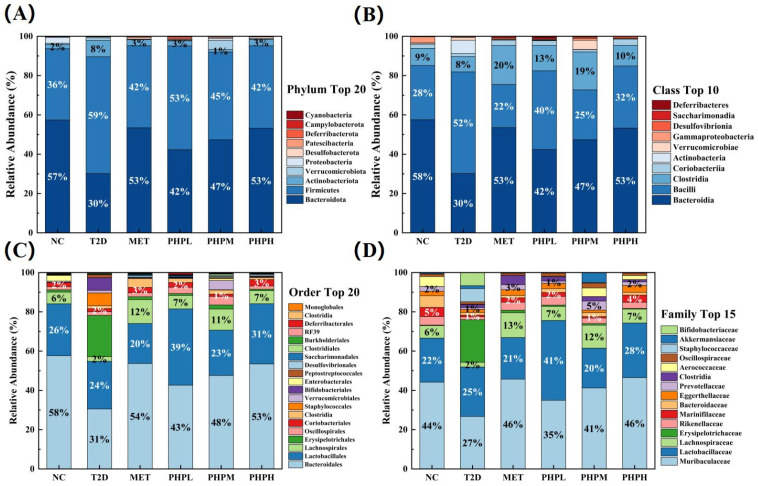
PHP affects intestinal species composition in T2Dm mice: (**A**) relative abundance of phyla; (**B**) relative abundance of classes; (**C**) relative abundance of orders; (**D**) relative abundance of families.

Gut microbiota exerts dual effects on type 2 diabetes (T2DM): under different conditions, it may either exacerbate T2DM progression or exert a therapeutic effect, and these two opposing effects are mediated through regulating short-chain fatty acids (SCFAs), inflammation, bile acid metabolism, and energy homeostasis. Species composition analysis enables multilevel evaluation of PHP’s hypoglycemic effects from a microbiome perspective. As shown in Figure 6A, the Firmicutes-to-Bacteroidetes (F/B) ratio at the phylum level was significantly elevated in the T2DM group compared to the NC group—a pattern typical of obesity/diabetes and positively correlated with insulin resistance [70]. All PHP dose groups exhibited significant dose-dependent decreases in F/B values, driven primarily by increased abundance of Bacteroidetes—a phylum that produces short-chain fatty acids (SCFAs, e.g., acetic/butyric acid) to regulate glycolipid metabolism. The PHPH group exhibited no significant difference in the F/B ratio compared to the MET group. Since the F/B ratio correlates with intestinal energy metabolism and gut barrier integrity [71], this result suggests PHP modulates gut microbiota structure similarly to MET. Such modulation may help ameliorate hyperglycemia, though specific pathways (e.g., reduced energy absorption and improved gut barrier function) need direct experimental verification. As shown in Figure 6B, at the class level, the T2DM group exhibited a significant reduction in the abundance of Bacteroidia (phylum Bacteroidetes)—a change that may contribute to decreased peripheral insulin sensitivity. Concomitantly, the group showed a marked increase in the abundance of Bacilli (phylum Firmicutes), a shift that promotes excessive lactic acid production via fermentation. This lactic acid overproduction may stimulate intestinal secretion of 5-hydroxytryptamine (5-HT), which in turn indirectly inhibits insulin release [72]. Like the MET group, the PHPM group significantly increased the abundance of Clostridia (phylum Firmicutes). Clostridia produce short-chain fatty acids (SCFAs, e.g., butyric acid), which enhance intestinal barrier integrity, suppress inflammation, and regulate metabolism [73]. Compared to the T2DM group, the PHPM group also exhibited a 50% reduction in Bacilli abundance, suggesting potential amelioration of D-lactate-induced metabolic acidosis.

As shown in Figure 6C, at the order level, the T2DM group exhibited a significant increase in Erysipelotrichia (phylum Firmicutes)—a change that may promote fat absorption, exacerbate obesity, and induce inflammatory factor release. Concomitantly, the group showed a marked reduction in Bacteroidales (phylum Bacteroidetes), which could potentially accelerate Th17-mediated inflammatory responses [74]. Compared to T2DM mice, the PHPH group exhibited a 96.60% decrease in Erysipelotrichia and a 76.43% increase in Bacteroidales, suggesting PHP may mitigate insulin resistance via immune modulation. As shown in Figure 6D, at the family level, the T2DM group had significantly reduced abundances of Muribaculaceae and Lachnospiraceae, while the PHP group showed upregulated abundances of these two families. This observation suggests PHP may either activate the insulin receptor/phosphatidylinositol-3-kinase/protein kinase B (IRS/PI3K/Akt) signaling pathway [75] or inhibit inflammation by modulating the Th17/Treg balance.

#### 3.9.2. α-Diversity and β-Diversity

**Figure 7:**
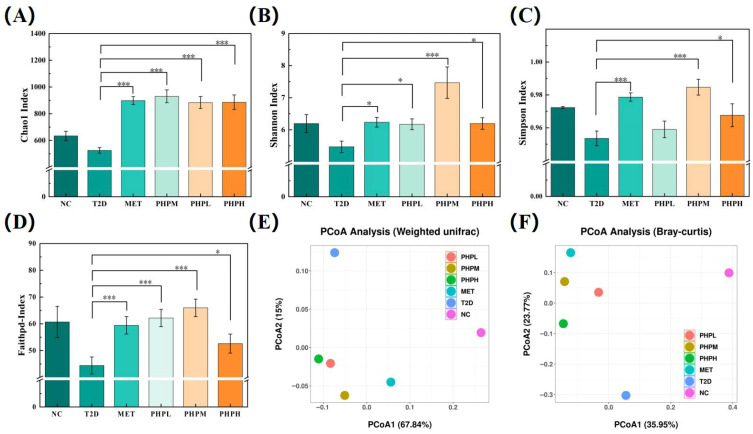
Effects of PHP on the α- and β-diversity of intestinal flora in T2DM mice: (**A**) Chao1 index; (**B**) Shannon index; (**C**) Simpson index; (**D**) Faith PD index; (**E**) principal coordinates analysis (weighted unifrace); (**F**) principal coordinates analysis (Bray–Curtis). Difference was significant at * *p* < 0.05, *** *p* < 0.001.

A-Diversity quantifies species richness and evenness within a localized habitat. Good’s coverage indices were ≥0.999 across all samples, confirming sufficient sequencing depth for analysis. As shown in Figure 7A–D, the T2DM group exhibited a significant reduction in α-diversity compared to NC mice (Chao1: −17.06%; Simpson: −1.93%; Shannon: −11.70%; Faith PD: −26.67%)—a pattern typical of diabetic mouse models [76]. In T2DM mice, the PHPM group demonstrated the most robust restoration of intestinal flora α-diversity (Chao1: +77.14%; Simpson: +3.27%; Shannon: +36.57%; Faith PD: +48.49%) and significantly outperformed several natural product interventions [30,76].

Β-Diversity measures dissimilarity in community composition across habitats. Here, we used a principal coordinates analysis (PCoA) to assess intergroup heterogeneity in intestinal flora structure. As shown in Figure 7E, the T2DM group formed a triangular distribution relative to the NC and MET groups in the 2D ordination space, indicating significant structural differentiation. The PHP and MET groups exhibited highly overlapping distributions, both distant from the T2DM cluster, suggesting that PHP may exert hypoglycemic effects by targeting microbial taxa similar to those targeted by MET [76]. As shown in Figure 7F, the first two PCoA axes (PCoA1 + PCoA2) explained 50.12% of cumulative variance, confirming significant intergroup differences in species abundance distribution.

#### 3.9.3. Species Differential Analysis and Signature Taxa

**Figure 8:**
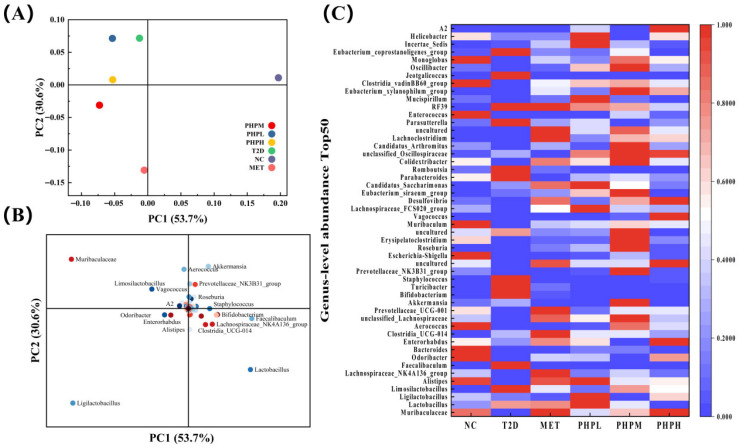
Species differences and signature species: (**A**) principal component analysis between groups; (**B**) principal component analysis of species; (**C**) heat map of genus-level composition for species clustering.

Principal component analysis (PCA) quantifies inter-sample differences in species composition to identify drivers of abundance variation. As shown in Figure 8A, the T2DM and MET groups exhibited strongly significant opposing environmental preferences: the T2DM group showed positive loading on the PC2 axis, while the MET group showed negative loading. Figure 8B reveals that the PHP group upregulated *Muribaculaceae*, *Limosilactobacillus*, and *Ligilactobacillus*—taxa that contributed significantly to inter-sample differentiation. *Limosilactobacillus* and *Ligilactobacillus* are beneficial lactobacilli that ameliorate type 2 diabetes by reducing oxidative stress and inflammation [77]. Changes in *Muribaculaceae* abundance, analogous to drug-treated states, enhance insulin sensitivity and regulate glycemia via short-chain fatty acid (SCFA) production.

As shown in Figure 8C, analysis of the top 50 genera ranked by average abundance revealed increased levels of *Limosilactobacillus*, *Bifidobacterium*, and *Parabacteroides* in T2DM mice. These taxa have been linked to diabetes-induced chronic inflammation [78] and heightened susceptibility to inflammatory bowel disease [79], potentially reflecting host-driven immune homeostasis via upregulated anti-inflammatory taxa. PHP significantly reduced intestinal pathogenic bacteria in T2DM mice (*Turicibacter*: −54.80%; *Staphylococcus*: −30.91%), alleviating pathogen-induced adipose tissue inflammation, insulin resistance, and bile acid dysmetabolism [80]. Notably, six taxa (*Alistipes*, *Romboutsia*, *Parasutterella*, *Clostridia vadinBB60*, *Jeotgalicoccus*, and *Eubacterium coprostanoligenes*) that exhibited T2DM-induced increases in abundance normalized with PHP, suggesting their potential roles in T2DM regulation—though specific mechanisms remain undefined.

#### 3.9.4. Metabolic Pathway and Functional Annotation Analysis

**Figure 9:**
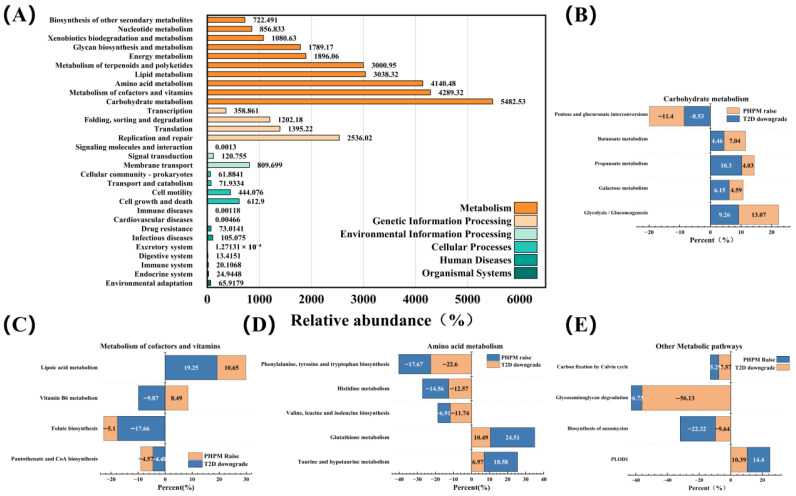
(**A**) Predicted KEGG secondary functional pathway abundance map; (**B**) significantly altered Kos in carbohydrate metabolism; (**C**) significantly altered Kos in metabolism of cofactors and vitamins; (**D**) significantly altered Kos in amino acid metabolism; (**E**) significantly altered Kos in other metabolism pathways.

The KEGG pathway database (https://www.kegg.jp/) classifies metabolic networks into six primary categories: Metabolism, Genetic Information Processing, Environmental Information Processing, Cellular Processes, Organismal Systems, and Human Diseases. Each category is hierarchically structured into four levels: 45 subfunctional modules (Level II), pathway maps (Level III), and KEGG Orthology (KO) annotations (Level IV) [81]. As shown in Figure 9A, this study identified 30 KEGG Level II functional pathways, with the top five most abundant pathways all categorized under metabolism: carbohydrate metabolism, cofactor/vitamin metabolism, amino acid metabolism, lipid metabolism, and terpenoid/polyketide metabolism. A total of 159 KOs were detected across six sample groups, 131 of which were shared among the T2DM, NC, and PHPM groups. High-abundance pathways from these 131 KOs were selected for analysis. Specifically, 20 KOs in the PHPM group with abundance patterns opposite to those in T2DM and similar to those in MET were shortlisted, of which 17 metabolic pathway KOs were subjected to detailed analysis.

As shown in Figure 9B, within the carbohydrate metabolic network, PHP regulated metabolic dysfunctions in T2DM mice primarily by upregulating pathway abundances of pentose/glucuronate interconversion (involved in carbohydrate recycling and glucose homeostasis), glycolysis/gluconeogenesis (central to glucose breakdown and synthesis), and butanoate metabolism (linked to intestinal environment modulation). The KEGG pathway changes induced by PHP resemble those of canagliflozin (a recognized intestinal glucose absorption modulator) [82] and astragalus polysaccharide (reported to regulate intestinal glucose transporters) [83], supporting the inference that PHP may ameliorate metabolic disorders by modulating intestinal glucose absorption.

As shown in Figure 9C,D, PHP upregulated cofactor/vitamin metabolism and amino acid metabolism pathways, which may contribute to hypoglycaemic effects. Vitamin B6 is a precursor for pyridoxal phosphate (PLP): PLP regulates hepatic gluconeogenesis/glycolysis and improves insulin resistance via isoleucine/leucine metabolism, and as a coenzyme precursor in glutathione metabolism, it works with α-lipoic acid (ALA) to alleviate diabetic oxidative stress and inflammation [84]. ALA may reduce oxidative damage in pancreatic β-cells/hepatocytes (via Nrf2 pathway activation), mitigate adipose tissue inflammation (by promoting M1-to-M2 macrophage polarization [85]), and improve insulin resistance (via AMPK signaling activation); it may also inhibit glucose-6-phosphatase (G6P) via AMPK/mTOR pathway regulation [86]. Additionally, PLP and ALA may co-regulate tryptophan biosynthesis to promote indole production, activating aryl hydrocarbon receptors [87] and enhancing intestinal barrier function. The upregulated folate biosynthesis pathway likely results from increased Lactobacillus abundance (restoring intestinal homeostasis [88]), consistent with the Section 3.9.3 findings.

As shown in Figure 9E, the significant upregulation of the glycosaminoglycan degradation pathway further supports PHP’s anti-inflammatory effects and its ability to alleviate insulin resistance. Cross-validation with the MetaCyc database (https://metacyc.org/) revealed that the five most abundant pathways—nucleoside/nucleotide biosynthesis, amine/polyamine degradation, cofactor/prosthetic group/electron carrier/vitamin biosynthesis, fatty acid/lipid biosynthesis, and fermentation—exhibited high overlap with KEGG annotations, underscoring the consistency of predictive outcomes.

## 4. Conclusions

We extracted, purified, and characterized the peptide (PHP) from *Pinus pumila*. PHP inhibits α-amylase and α-glucosidase. In T2DM mice, it improves basal physiological metabolic indicators, glucose homeostasis, and organ morphology; modulates lipids; activates glycolysis; suppresses the compensatory activation of the pentose phosphate pathway to ameliorate insulin resistance; and strengthens intestinal barrier function by restoring gut microbiota diversity. KEGG analysis suggests that the multi-dimensional glucose-lowering effects may be closely associated with AMPK regulation, inflammation inhibition, and enhanced insulin sensitivity; we will focus on verifying these mechanisms in PHP in future studies. PHPH occasionally shows suboptimal efficacy vs. PHPM, highlighting the need for precise PHP dosing. While PHP has high hypoglycemic activity among natural peptides, it lags behind anti-diabetic drugs and is most promising as a dietary supplement. PHP production generates no organic wastewater or bioaccumulative toxic by-products, promotes agricultural by-product reuse, and benefits health. Its short half-life and poor bioavailability currently limit translation to nutraceuticals, though emerging strategies (microencapsulation, nanoparticle delivery, and permeation enhancers) address these issues. This work establishes a paradigm for high-value utilization of *Pinus pumila* oil meal and provides a scientific basis for developing natural hypoglycaemic functional foods.

## Figures and Tables

**Table 1:** In vitro inhibitory activity of peptides during purification and digestion.

Purification Stage	α-Amylase IC_50_ (mg mL^−1^)	*p*-Value	α-Glucosidase IC_50_ (mg mL^−1^)	*p*-Value
Primary purification		*p* < 0.001 ***		*p* < 0.001 ***
H_2_O	20.45 ± 3.26		40.89 ± 5.77	
0.1 M NaCl	23.42 ± 2.72	*	31.461 ± 3.58	**
0.3 M NaCl	25.91 ± 2.43	**	24.89 ± 3.61	***
0.5 M NaCl	42.98 ± 5.34	***	46.71 ± 2.89	
Secondary purification		*p* = 0.009 **		*p* = 0.002 **
H_2_O (Fraction 1)	30.42 ± 4.20		27.08 ± 2.94	
H_2_O (Fraction 2)	20.72 ± 3.94	**	16.72 ± 2.88	**
Simulated gastric digestion				
PHP	1.71 ± 0.08		5.21 ± 0.14	

Note: * *p* < 0.05, ** *p* < 0.01, *** *p* < 0.001. For comparisons, we used H_2_O as the control group in the Primary purification stage and Fraction 1 as the control group in the Secondary purification stage; these comparisons were determined by one-way ANOVA followed by Dunnett’s post hoc test; *n* = 3.

**Table 2:** LC-MS/MS sequence identification results.

Fraction 2	Intensity (×10^7^)	PHP	Intensity (×10^7^)
GREEEEEAEERAA	121.87	NTDVQKLEHIFGAH	2871
SSERRGEEEDEDSSQK	4.988	NTDVQKLEHIFGAHR	191.53
SDDVLEAAFNTDVQKLEHIFGAH	2056.7	FNTDVQKLEHIFGAH	287.24
ALPNFGEVSELLEGISRY	56.311	KLEEGDVFGVPSGHT	12.68
RGREEEEEAEERAA	206.75	KLEEGDVFGVPSGHTF	6.0224
ELLEGI	64.932	NTDVQKLEHIFGAHRRGVIF	473.87
GPKDNPFLDSVDVT	10.735	STSASEQPKPFNL	17.621
LPNFGEVSELLEGISRY	3.959	EYEPFYVAGGRNPETVY	11.331
EEEEDSSQKVR	16.893	KLEEGDVFGVPSGHTFY	4.9454
RGREEEEEAEERA	80.938	FNTDVQKLEHIF	7.5018

## Data Availability

This article contains all experimental data supporting the results of this study. Detailed study protocols and parameter descriptions are available from the corresponding author upon reasonable request.

## Supplementary Materials

The following supporting information can be downloaded at: https://www.mdpi.com/article/10.3390/nu17172903/s1, Table S1:Complete LC-MS/MS Identification Results of Fraction 2 and PHP.
